# Preoperative hemoglobin glycation index and postoperative complications after non-cardiac surgery: a retrospective cohort study

**DOI:** 10.1007/s44254-026-00179-w

**Published:** 2026-06-22

**Authors:** Yi Zhang, Ling Lan, Yuelun Zhang, Le Shen

**Affiliations:** 1 Department of Anesthesiology, Peking Union Medical College Hospital, Chinese Academy of Medical Sciences and Peking Union Medical College, Beijing, 100730 China; 2 Chinese Academy of Medical Sciences and Peking Union Medical College, Beijing, 100730 China; 3 Medical Research Center, Peking Union Medical College Hospital, Chinese Academy of Medical Sciences and Peking Union Medical College, Beijing, 100730 China

**Keywords:** Hemoglobin glycation index, Non-cardiac surgery, Postoperative complications

## Abstract

**Purpose:**

Perioperative metabolic vulnerability is an important contributor to postoperative morbidity, yet preoperative risk stratification relies largely on conventional glycemic measures such as hemoglobin A1c (HbA1c) and fasting plasma glucose (FPG). Hemoglobin glycation index (HGI) quantifies interindividual discordance between HbA1c and contemporaneous glycemia and may capture metabolic phenotypes not reflected by absolute glucose values. We investigated the association between preoperative HGI and postoperative outcomes in adults undergoing non-cardiac surgery.

**Methods:**

We conducted a retrospective single-center cohort study including adult inpatients who underwent non-cardiac surgery under general anesthesia between January 2013 and June 2024. Patients with both preoperative HbA1c and FPG measured within 60 days before surgery were included. HGI was calculated as the residual of observed HbA1c minus HbA1c predicted from FPG using a cohort-specific linear regression model. The primary outcome was any postoperative complication occurring before hospital discharge. Secondary outcomes included major complications (Clavien–Dindo grade ≥ III), organ-specific complications, deep-vein thrombosis, Intensive Care Unit (ICU) admission, hospital and postoperative length of stay, and in-hospital mortality. Associations were evaluated using multivariable regression models with HGI analyzed as a continuous variable (per 1–SD increase) and by quartiles (Q2 as reference), complemented by restricted cubic spline analyses and prespecified subgroup analyses.

**Results:**

A total of 24,307 patients were included (mean age 58.8 ± 13.3 years; 51% women). Postoperative complications occurred in 8.4% of patients. In adjusted continuous models, higher HGI was independently associated with postoperative complications (odds ratio [OR] per 1–SD increase 1.068; 95% confidence interval [CI], 1.018–1.120; *P* = 0.007), urinary complications (OR 1.155; 95% CI, 1.024–1.301; *P* = 0.018), and longer total hospital length of stay (adjusted mean ratio 1.030; 95% CI, 1.017–1.043;* P* = 0.001). Quartile analyses demonstrated the lowest risk of postoperative complications in the mid-range HGI group, with higher risks observed at extreme HGI values. Restricted cubic spline models revealed an approximately linear association between HGI and overall postoperative complications, while outcome-specific nonlinear relationships were observed for major complications, ICU admission, and in-hospital mortality. Associations were generally consistent across subgroups, with no statistically significant interaction detected across prespecified strata.

**Conclusions:**

Preoperative hemoglobin glycation index was independently associated with postoperative complications after non-cardiac surgery and identified nonlinear risk patterns for selected severe outcomes. HGI may serve as a complementary perioperative metabolic risk marker beyond HbA1c and fasting plasma glucose to provide incremental risk information.

**Supplementary Information:**

The online version contains supplementary material available at 10.1007/s44254-026-00179-w.

## Introduction

Postoperative complications are common and clinically significant. A systematic review reported that 14.4% of surgical patients experienced an adverse event, of which 5.2% were considered potentially preventable [1]. Among in-hospital adverse events, up to 39.6% are surgery-related [2]. These complications are frequently encountered and are associated with prolonged hospital stay and increased healthcare costs. Rigorous, comprehensive assessment of risk factors can help clinicians maintain vigilance and may prevent such complications.

Perioperative hyperglycemia is common among patients undergoing non-cardiac surgery and has been associated with adverse postoperative outcomes, including surgical-site infection, myocardial injury, and delayed recovery [3–6]. Hemoglobin A1c (HbA1c) is an established measure of chronic glycemia and a surrogate endpoint in trials of glucose-lowering interventions [7]. It is strongly associated with the risk of diabetes-related complications [8]. Nevertheless, despite its widespread use to guide and monitor diabetes care, HbA1c has important limitations in clinical practice. Inter-individual differences in glycation propensity—observed in both individuals with and without diabetes—may limit the utility of HbA1c as a one-size-fits-all metric [9–12]. The hemoglobin glycation index (HGI) quantifies the deviation between an individual’s observed HbA1c and the value predicted from fasting plasma glucose (FPG) using a linear regression model; in other words, HGI = observed HbA1c − predicted HbA1c (FPG), i.e., the residual from the fitted regression line [13, 14]. By capturing this residual, HGI can help mitigate the influence of short-term glycemic fluctuations on HbA1c and better characterize individual glycemic profiles [15] Prior studies suggest that HGI is approximately normally distributed, stable over time, and consistent across a wide range of glucose concentrations [16–18].

Although HGI has been investigated mainly in patients with diabetes and in relation to chronic microvascular or cardiovascular outcomes, its relevance in the perioperative setting remains poorly understood. In particular, evidence is lacking on whether preoperative HGI provides prognostic information in adults undergoing non-cardiac surgery, beyond conventional glycemic indicators such as HbA1c and fasting plasma glucose. Moreover, few data are available regarding its association with clinically important postoperative endpoints, including severe complications and organ-specific complications.

Therefore, we conducted a retrospective cohort study to examine the association between preoperative HGI and postoperative outcomes in adults undergoing non-cardiac surgery. We specifically evaluated whether HGI was associated not only with overall postoperative complications, but also with severe complications and organ-specific complications. We hypothesized that higher HGI, reflecting greater glycation propensity at a given fasting plasma glucose level, would be independently associated with an increased risk of adverse postoperative outcomes.

## Methods

### Study design and data sources

This retrospective, single-center cohort study was conducted at Peking Union Medical College Hospital (PUMCH), a large tertiary-care academic medical center. We extracted data from two institutional sources—the Clinical Data Repository and the PeriOperative Patient Safety database—covering January 2013 through June 2024. The study protocol was approved by the PUMCH Institutional Review Board (I-24PJ1584) with trial registration as ChiCTR2500104768, which granted a waiver of informed consent owing to the retrospective design.

### Study population

We identified adult inpatients (≥ 18 years) who underwent non-cardiac surgery under general anesthesia during the study period. Patients were excluded if they (1) lacked either preoperative HbA1c or FPG measurements within 60 days before surgery; (2) had a documented hemoglobinopathy or received a blood transfusion within the preceding 2 months; or (3) underwent obstetric procedures. After applying these criteria, 24,307 patients were included in the final analytic cohort. Transplant procedures were not included in this cohort. At our institution, transplant surgeries are performed infrequently and were not identified in the study dataset based on procedural coding.

### Study outcomes

The primary outcome was any postoperative complication occurring after the operation and before hospital discharge, as recorded in the discharge diagnoses, based on International Classification of Diseases (ICD)-coded diagnoses and structured discharge records. Secondary outcomes comprised: (1) major complications, defined as Clavien–Dindo grade ≥ III [19]; (2) organ-specific complications (pulmonary, abdominal, urinary, cardiac, neurological, infectious); (3) deep-vein thrombosis (DVT); (4) Intensive Care Unit (ICU) admission; (5) ICU length of stay (LoS); (6) total hospital length of stay and postoperative LoS; and (7) in-hospital mortality. Detailed definitions (and code lists, where applicable) for all secondary outcomes are provided in Supplementary Table 1.

### Definition of exposure variables

**Fig. 1:**
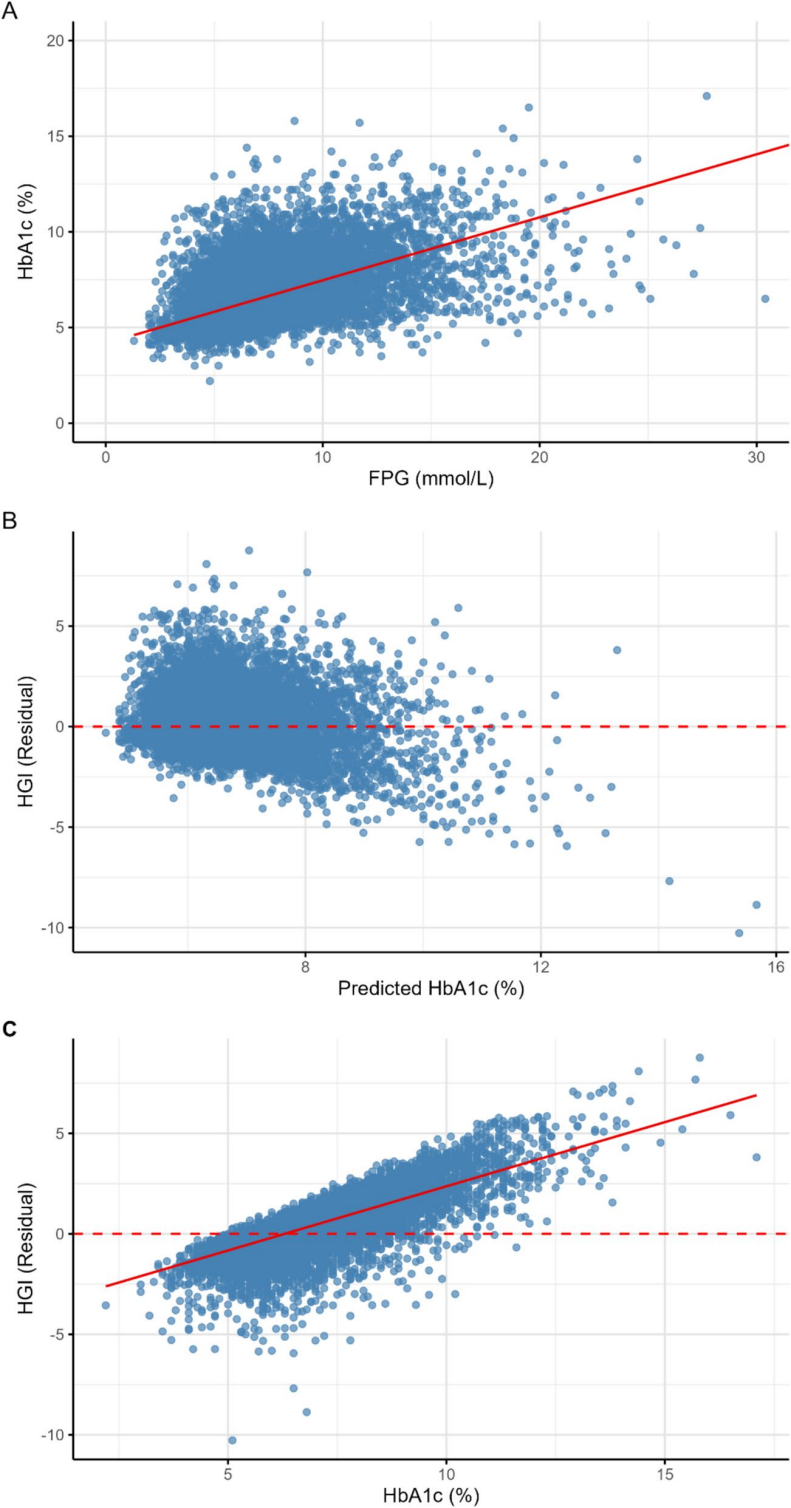
Derivation of the HGI. **A** Relationship between FPG and HbA1c. The dotted line represents the cohort-specific simple linear regression used to estimate predicted HbA1c: HbA1c (%) = 4.18 + 0.3293 × FPG (mmol/L) (*R*^*2*^ = 0.3618). **B** Distribution of HGI values plotted as residuals (observed minus predicted HbA1c) against predicted HbA1c. **C** Relationship between HGI (residuals) and observed HbA1c. The horizontal dashed line indicates HGI = 0, with positive values denoting higher-than-expected HbA1c for a given FPG and negative values denoting lower-than-expected HbA1c. *FPG* fasting plasma glucose, *HbA1c* Hemoglobin A1c, *HGI *hemoglobin glycation index

The primary exposure was the preoperative HGI, defined for each patient as the difference between the observed HbA1c and the value predicted from FPG using a cohort-specific linear regression model—that is, the residual from the fitted regression. Before model derivation, we excluded 649 patients with missing preoperative HbA1c (n = 324) or FPG (n = 325), leaving 24,307 patients in the analytic dataset. In this sample, the relationship between baseline HbA1c and FPG was described by the ordinary least-squares model: HbA1c (%) = 4.18 + 0.3293 × FPG (mmol/L) (*R*^*2*^ = 0.3618, Fig. 1A). Predicted HbA1c for each patient was obtained by inserting their FPG into this equation, and HGI was then calculated as HGI = observed HbA1c − predicted HbA1c (Fig. 1B). To assess model adequacy, we visually examined the HbA1c–FPG relationship and compared the linear model with a spline-based specification. Although the spline model may provide a better statistical fit, we adopted a linear regression model for HGI derivation to maintain consistency with prior literature. where HGI is defined as a linear residual-based measure. We retained the linear model for HGI derivation to preserve consistency with the conventional definition of HGI and to ensure interpretability of the regression equation. By construction, a positive HGI denotes higher-than-expected HbA1c for a given FPG (greater glycation propensity), whereas a negative HGI denotes lower-than-expected HbA1c. The correlation between HGI and HbA1c is shown in Fig. 1C.

### Covariates and data collection

Demographic, laboratory, and perioperative data were extracted from institutional electronic medical records. Covariates were defined using information available prior to or during surgery, whereas postoperative data were used only for outcome ascertainment. HbA1c testing is routinely performed and standardized to the National Glycohemoglobin Standardization Program, with results reported as percentages. Preoperative FPG is also routinely measured in surgical candidates using venous samples. Preoperative HbA1c and FPG were defined as measurements obtained within 60 days before surgery; when multiple values were available, the value closest to the index operation was used. The index operation was defined as the first eligible surgery during the study period for each patient. Patient characteristics included age, sex, body mass index (BMI), American Society of Anesthesiologists (ASA) physical status, smoking status, and alcohol use. Surgical covariates included procedural urgency, operative duration, estimated blood loss, and surgical type. Procedures were grouped into nine categories—neurosurgical, vascular, orthopedic, head and neck, urological, thoracic, gynecologic, abdominal, and other—based on procedure codes and institutional surgical catalogs, with category definitions provided in Supplementary Table 2. Comorbidity burden was quantified using the van Walraven–weighted Elixhauser comorbidity score, derived from ICD-coded diagnoses recorded prior to the index surgery and calculated with the R package comorbidity. In addition, several prespecified clinically relevant conditions (hypertension, coronary artery disease, cerebrovascular disease, chronic kidney disease, chronic obstructive pulmonary disease, hyperlipidemia, and malignant tumor) were also ascertained from diagnosis codes and included as separate covariates for clinical interpretability and because some conditions are not explicitly captured as individual Elixhauser categories in the weighted score. Diabetes status was defined based on documented clinical diagnosis; information on disease duration and treatment details was not available.

### Statistical analysis

HGI was categorized into quartiles based on its distribution in the analytic cohort: Q1 (n = 6,077, HGI ≤ − 0.53), Q2 (n = 6,077, − 0.53 < HGI ≤ − 0.17), Q3 (n = 6,086, − 0.17 < HGI ≤ 0.35), and Q4 (n = 6,067, HGI > 0.35). Baseline characteristics across HGI quartiles were compared using one-way analysis of variance or Kruskal–Wallis tests for continuous variables, as appropriate, and χ^2^ or Fisher’s exact tests for categorical variables. Continuous variables are presented as mean (standard deviation [SD]) or median (interquartile range [IQR]), and categorical variables as number (percentage).

An a priori sample-size calculation was performed in PASS 2021 (NCSS LLC, Kaysville, UT, USA). Assuming a baseline incidence of postoperative complications of 12% (*P₀* = 0.12) [20–23], and a clinically meaningful odds ratio (OR) of 1.08 per 1-SD increase in HGI, a sample size of 20,999 patients would provide 90% power with a two-sided *α* = 0.05, allowing for covariates explaining up to 20% of outcome variance (*R*^*2*^ = 0.20).

Multivariable logistic regression models were fitted to estimate the association between HGI and the primary outcome, adjusting for prespecified demographic factors, comorbidities, and surgical characteristics, including the van Walraven–weighted Elixhauser comorbidity score. HGI was modeled (i) as a continuous variable (per 1-SD increase) and (ii) as a categorical variable using quartiles to facilitate clinical interpretability and to complement nonlinear modeling. Quartiles were defined a priori, and Q2 was selected as the reference group because it represents a mild negative (near-neutral) glycation phenotype and approximates a clinically stable range of HGI. Multicollinearity among covariates (including the weighted Elixhauser score and individual comorbidity indicators) was evaluated using variance inflation factors (VIFs); no problematic multicollinearity was observed (all VIFs < 2, Supplementary Table 3), and all prespecified covariates were retained in the final models. Potential nonlinearity in the continuous HGI–outcome relationship was examined using restricted cubic spline (RCS) functions. In the primary spline analyses, HGI was modeled using 3 knots, which was prespecified for parsimony and model stability across outcomes. When only the number of knots is specified in the rms package, knot locations are generated automatically from the empirical distribution of the predictor according to Harrell’s recommended quantiles; for 3-knot models, these correspond approximately to the 10th, 50th, and 90th percentiles of the HGI distribution. We used a common 3-knot specification across outcomes to enhance comparability and avoid outcome-specific data-driven overfitting. In quartile-based models, tests for linear trend across quartiles (treating quartiles as an ordinal variable) and for departure from linearity (quadratic term) were additionally performed.

Missing covariate data were handled using multiple imputation by chained equations (MICE; mice package in R, version 3.17.0) [24]. Five imputed datasets (m = 5) were generated with 50 iterations per dataset to ensure convergence. Continuous, binary, and categorical variables were imputed using predictive mean matching (“pmm”), logistic regression (“logreg”), and polytomous regression (“polyreg”), respectively. Given the retrospective design and the absence of evidence suggesting systematic missingness related to unobserved outcomes, the missing data mechanism was assumed to be missing at random (MAR), conditional on the observed covariates included in the imputation model. All variables used in the analytic models were included in the imputation procedure to improve plausibility of the MAR assumption. Estimates across imputed datasets were combined using Rubin’s rules.

Secondary outcomes were analyzed using logistic regression for binary outcomes (e.g., major complications, ICU admission, organ-specific complications, DVT, and in-hospital mortality). For length-of-stay outcomes, generalized linear models were applied under distributional assumptions appropriate for right-skewed data. ICU length of stay was analyzed among patients admitted to the ICU. Prespecified subgroup analyses were conducted by sex, age (< 60 vs ≥ 60 years), HbA1c (< 6.5% vs ≥ 6.5%), ASA physical status (I–II vs III–V), diabetes, and surgical urgency (elective vs emergent). To further assess the reliability of interaction tests, we performed post-hoc power analyses for each subgroup-specific interaction using both Wald-based approximation and simulation-based methods. These analyses were intended to evaluate whether the study had sufficient statistical power to detect potential effect modification across subgroups. No adjustment for multiple comparisons was performed; therefore, results from secondary, subgroup, and sensitivity analyses should be interpreted as exploratory. This approach was chosen because these analyses were prespecified and intended to explore potential heterogeneity and generate hypotheses rather than to provide confirmatory inference. All analyses were conducted in R version 4.4.1 (R Foundation for Statistical Computing, Vienna, Austria). Two-sided *P* values < 0.05 were considered statistically significant.

As a sensitivity analysis, glycemic status was reclassified into three categories based on documented diagnosis and laboratory criteria: (1) documented diabetes; (2) possible undiagnosed diabetes, defined as the absence of a documented diagnosis but meeting laboratory criteria for diabetes (HbA1c ≥ 6.5% and/or fasting plasma glucose ≥ 7.0 mmol/L); and (3) no evidence of diabetes. Multivariable models were repeated using this 3-level variable in place of the original binary diabetes covariate.

## Results

### Study population and baseline characteristics

**Fig. 2:**
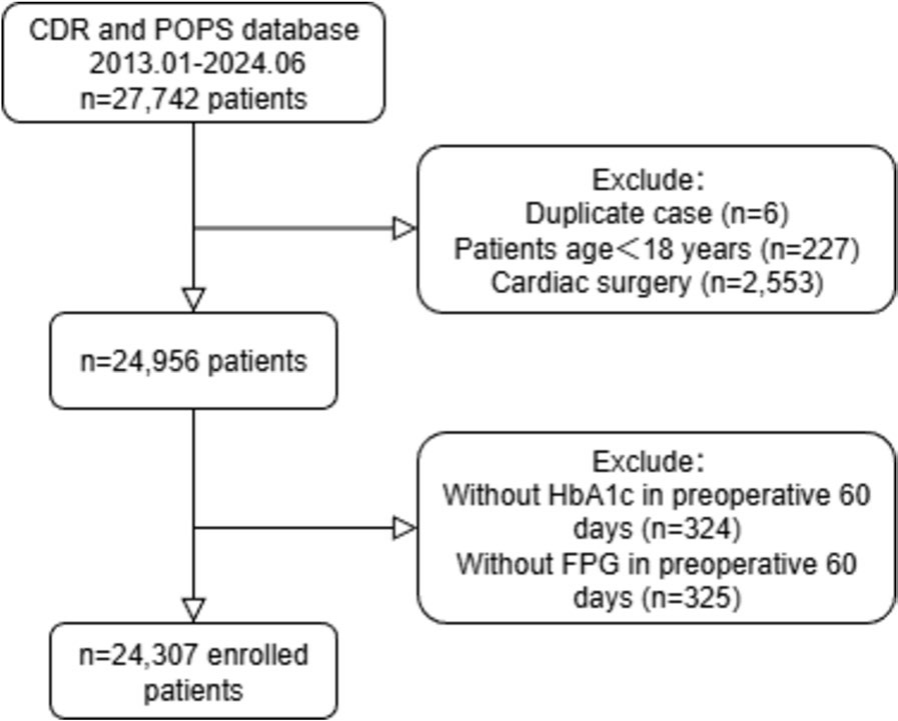
Flow diagram of patient selection. A total of 27,742 adult patients with preoperative HbA1c data were identified from the Clinical Data Repository (CDR) and PeriOperative Patient Safety (POPS) databases between January 2013 and June 2024. After exclusion of duplicate records (n = 6), patients aged < 18 years (n = 227), cardiac surgery cases (n = 2,553), patients without valid preoperative HbA1c measurements within 60 days before surgery (n = 324), and those without valid fasting plasma glucose (FPG) measurements within 60 days (n = 325), 24,307 patients undergoing non-cardiac surgery under general anesthesia were included in the final analytic cohort

Between January 2013 and June 2024, 27,742 adult surgical patients were screened, and 24,307 patients undergoing non-cardiac surgery under general anesthesia were included in the final analytic cohort (Fig. 2). Baseline characteristics stratified by HGI quartiles are presented in Table 1. The mean age was 58.79 ± 13.29 years, and 51% were women. Across increasing HGI quartiles, patients tended to be older (54.88 ± 14.56 years in Q1 vs 61.77 ± 11.83 years in Q4) and have slightly higher BMI (24.57 ± 3.93 vs 25.53 ± 3.83). HbA1c increased stepwise from Q1 to Q4 (5.41 ± 0.74% to 7.86 ± 1.36%), whereas fasting plasma glucose showed a non-monotonic pattern (6.79 ± 3.06 mmol/L in Q1, 5.66 ± 1.48 mmol/L in Q2, 6.03 ± 1.72 mmol/L in Q3, and 7.26 ± 2.44 mmol/L in Q4), consistent with the residual-based definition of HGI. The prevalence of diabetes increased markedly across quartiles (23% in Q1, 22% in Q2, 43% in Q3, and 86% in Q4), and hypertension became more common from Q1 to Q4 (40% to 60%). Emergency surgery was most frequent in Q1 (5.7%) compared with Q2–Q4 (1.9–3.3%), and the proportion of ASA physical status III–V increased from 18% in Q1 to 28% in Q4.

**Table 1:** Baseline characteristics of patients undergoing non-cardiac surgery according to hemoglobin glycation index quartiles

**Characteristic**	Overall	Q1 (Lowest)	Q2 (Low)	Q3 (High)	Q4 (Highest)	Missing data
	*n* = 24,307	*n* = 6,077	*n* = 6,077	*n* = 6,086	*n* = 6,067	
**Patient Factors**						
Age, years, mean (SD)	58.79 (13.29)	54.88 (14.56)	57.35 (13.34)	61.16 (12.03)	61.77 (11.83)	
Sex, n (%)						
Women	12,449 (51%)	2,867 (47%)	3,002 (49%)	3,149 (52%)	2,840 (47%)	
Men	11,858 (49%)	3,210 (53%)	3,075 (51%)	2,937 (48%)	3,227 (53%)	
BMI, mean (SD)	25.17 (3.88)	24.57 (3.93)	25.08 (3.88)	25.51 (3.78)	25.53 (3.83)	340
HGI, mean (SD)	0.00(1.02)	−1.00 (0.62)	−0.35 (0.10)	0.06 (0.15)	1.29 (0.99)	
HbA1c, %, mean (SD)	6.29 (1.28)	5.41 (0.74)	5.69 (0.50)	6.22 (0.60)	7.86 (1.36)	
FPG, mmol/L, mean (SD)	6.44 (2.34)	6.79 (3.06)	5.66 (1.48)	6.03 (1.72)	7.26 (2.44)	
Alcohol, n (%)	5,593 (23%)	1,383 (23%)	1,329 (22%)	1,344 (22%)	1,537 (25%)	
Smoking, n (%)	5,850 (24%)	1,284 (21%)	1,312 (22%)	1,481 (24%)	1,773 (29%)	
**Surgery Factors**						
EBL, ml, mean (SD)	91.09 (291.62)	99.91 (309.25)	87.24 (331.64)	81.09 (249.53)	96.43 (269.83)	2,224
Surgery duration, hours, median (IQR)	1.62 (0.73, 2.66)	1.42 (0.42, 2.55)	1.52 (0.47, 2.50)	1.70 (0.91, 2.66)	1.82 (1.07, 2.88)	2,300
Emergency case, n (%)	718 (3.3%)	302 (5.7%)	104 (1.9%)	126 (2.3%)	186 (3.3%)	2,224
ASA, n (%)						2,229
I-II	17,650 (80%)	4,357 (82%)	4,741 (85%)	4,487 (80%)	4,065 (72%)	
III-V	4,428 (20%)	973 (18%)	807 (15%)	1,090 (20%)	1,558 (28%)	
**Type of surgery**						
Abdominal, n (%)	3,688 (15%)	1,045 (17%)	715 (12%)	833 (14%)	1,095 (18%)	
Orthopedic, n (%)	5,627 (23%)	1,105 (18%)	1,526 (25%)	1,650 (27%)	1,346 (22%)	
Urological, n (%)	2,416 (9.9%)	447 (7.4%)	517 (8.5%)	628 (10%)	824 (14%)	
Thoracic, n (%)	1,275 (5.2%)	260 (4.3%)	311 (5.1%)	340 (5.6%)	364 (6.0%)	
Gynecologic, n (%)	1,190 (4.9%)	343 (5.6%)	233 (3.8%)	278 (4.6%)	336 (5.5%)	
Vascular, n (%)	1,812 (7.5%)	318 (5.2%)	438 (7.2%)	540 (8.9%)	516 (8.5%)	
Head and Neck, n (%)	1,575 (6.5%)	363 (6.0%)	354 (5.8%)	370 (6.1%)	488 (8.0%)	
Neurosurgical, n (%)	1,653 (6.8%)	403 (6.6%)	375 (6.2%)	414 (6.8%)	461 (7.6%)	
Other*, n (%)	5,072 (21%)	1,793 (30%)	1,609 (26%)	1,033 (17%)	637 (10%)	
**Comorbidity**						
Elixhauser score, median (IQR)	2.00 (1.00, 3.00)	2.00 (1.00, 3.00)	2.00 (1.00, 3.00)	2.00 (1.00, 3.00)	3.00 (2.00, 3.00)	
Diabetes, n(%)	10,523 (43%)	1,379 (23%)	1,328 (22%)	2,592 (43%)	5,224 (86%)	
Hypertension, n (%)	11,842 (49%)	2,437 (40%)	2,539 (42%)	3,246 (53%)	3,620 (60%)	
Hyperlipidemia, n (%)	5,714 (24%)	1,285 (21%)	1,482 (24%)	1,507 (25%)	1,440 (24%)	
Cerebrovascular disease, n (%)	1,974 (8.1%)	385 (6.3%)	364 (6.0%)	547 (9.0%)	678 (11%)	
Malignant tumor, n (%)	7,932 (33%)	1,862 (31%)	1,670 (27%)	1,976 (32%)	2,424 (40%)	
Chronic kidney disease, n (%)	395 (1.6%)	121 (2.0%)	73 (1.2%)	88 (1.4%)	113 (1.9%)	
Coronary heart disease, n (%)	3,203 (13%)	544 (9.0%)	616 (10%)	926 (15%)	1,117 (18%)	
COPD, n (%)	216 (0.9%)	50 (0.8%)	48 (0.8%)	68 (1.1%)	50 (0.8%)	

Values are presented as mean ± standard deviation, median (interquartile range), or number (percentage), as appropriate. HGI quartiles were defined as follows: Q1 (HGI ≤ − 0.53), Q2 (−0.53 < HGI ≤ − 0.17), Q3 (−0.17 < HGI ≤ 0.35), and Q4 (HGI > 0.35). ASA indicates American Society of Anesthesiologists physical status. The Elixhauser comorbidity score represents the van Walraven–weighted index derived from preoperative ICD-coded diagnoses. *P* values were calculated using analysis of variance or Kruskal–Wallis tests for continuous variables and χ^2^ tests for categorical variables

*ASA* American Society of Anesthesiologists, *BMI* body mass index, *COPD* chronic obstructive pulmonary disease, *EBL* estimated blood loss, *FPG* fasting plasma glucose, *HGI* hemoglobin glycation index, *HbA1c* Hemoglobin A1c, *ICD* International Classification of Diseases, *IQR* interquartile range, *SD* standard deviation

### Unadjusted postoperative outcomes by HGI quartiles

Clinical outcomes by HGI quartiles are summarized in Table 2. The overall incidence of postoperative complications (primary outcome) was 8.4% (2,048/24,307). The crude complication rate was lowest in Q2 (6.4%) and higher in both Q1 (10.0%) and Q4 (9.2%), suggesting increased risk at the extremes of the HGI distribution. A similar pattern was observed for major complications (Clavien–Dindo grade ≥ III), with rates of 4.7% in Q1 and 3.6% in Q4 compared with 2.2% in Q2. For organ-specific outcomes, higher crude rates in Q1 and/or Q4 were observed for pulmonary, abdominal, urinary, infectious, and cardiac complications, while deep vein thrombosis did not differ significantly across quartiles (*P* = 0.119). In-hospital mortality was highest in Q1 (1.7%) and lowest in Q2 and Q3 (both 0.2%). ICU admission occurred in 14% overall and was more frequent in Q1 and Q4 (both 17%) than in Q2 (10%). Both total hospital length of stay and postoperative length of stay differed across quartiles, with the longest stays observed in Q4.

**Table 2:** Unadjusted postoperative outcomes according to hemoglobin glycation index quartiles

**Characteristic**	Overall	Q1 (Lowest)	Q2 (Low)	Q3 (High)	Q4 (Highest)	*P* value
	*n* = 24,307	*n* = 6,077	*n* = 6,077	*n* = 6,086	*n* = 6,067	
**Primary outcome**						
Postoperative complications, n (%)	2,048 (8.4%)	634 (10%)	386 (6.4%)	468 (7.7%)	560 (9.2%)	0.001
**Secondary outcomes**						
Major complications, n (%)	774 (3.2%)	285 (4.7%)	131 (2.2%)	141 (2.3%)	217 (3.6%)	0.001
Pulmonary complications, n (%)	296 (1.2%)	114 (1.9%)	35 (0.6%)	60 (1.0%)	87 (1.4%)	0.001
Abdominal complications, n (%)	561 (2.3%)	212 (3.5%)	98 (1.6%)	111 (1.8%)	140 (2.3%)	0.001
Urinary complications, n (%)	180 (0.7%)	59 (1.0%)	22 (0.4%)	41 (0.7%)	58 (1.0%)	0.001
Infection complications, n (%)	545 (2.2%)	186 (3.1%)	81 (1.3%)	105 (1.7%)	173 (2.9%)	0.001
Cardiac complications, n (%)	228 (0.9%)	80 (1.3%)	41 (0.7%)	47 (0.8%)	60 (1.0%)	0.001
Neurological complications, n (%)	218 (0.9%)	71 (1.2%)	39 (0.6%)	51 (0.8%)	57 (0.9%)	0.020
Deep vein thrombosis, n (%)	248 (1.0%)	65 (1.1%)	49 (0.8%)	59 (1.0%)	75 (1.2%)	0.119
Mortality in hospital, n (%)	168 (0.7%)	102 (1.7%)	14 (0.2%)	15 (0.2%)	37 (0.6%)	0.001
ICU admission, n (%)	3,401 (14%)	1,030 (17%)	608 (10%)	719 (12%)	1,044 (17%)	0.001
ICU LoS, hours, mean (SD)*	12.67 (129.95)	23.59 (232.83)	6.03 (42.35)	7.11 (43.88)	13.97 (97.57)	0.001
LoS, days, median (IQR)	9.00 (5.00, 15.00)	9.00 (3.00, 16.00)	8.00 (3.00, 14.00)	9.00 (6.00, 15.00)	11.00 (7.00, 17.00)	0.001
Postoperative LoS, days, median (IQR)	4.71 (2.02, 7.63)	4.02 (0.95, 7.95)	3.89 (0.94, 6.81)	4.74 (2.72, 6.99)	5.43 (3.03, 7.97)	0.001

Values are presented as number (percentage) for categorical outcomes and median (interquartile range) for length-of-stay outcomes. Major complications were defined as Clavien–Dindo grade ≥ III. Postoperative complications were assessed from the end of surgery until hospital discharge. *P* values were calculated using χ^2^ tests for categorical outcomes and Kruskal–Wallis tests for continuous outcomes

*ICU* intensive care unit, *LoS* length of stay

### Multivariable associations between HGI and outcomes (continuous modeling)

Given the apparent excess risk at both extremes in crude analyses, we next quantified the independent association between HGI and outcomes using multivariable models. In adjusted analyses (covariates as specified in Methods, including the van Walraven–weighted Elixhauser comorbidity score), each 1-SD increase in HGI was associated with higher odds of postoperative complications (adjusted OR 1.068, 95% confidence interval [CI] 1.018–1.120; *P* = 0.007; Table 3). HGI was also associated with urinary complications (OR 1.155, 95% CI 1.024–1.301; *P* = 0.018) and deep vein thrombosis (OR 1.126, 95% CI 1.005–1.259; *P* = 0.039), while showing an inverse association with in-hospital mortality (OR 0.790, 95% CI 0.699–0.890; *P* = 0.001). For length-of-stay outcomes, higher HGI was associated with a longer total hospital stay (adjusted mean ratio [aMR] 1.030, 95% CI 1.017–1.043; *P* = 0.001), corresponding to an approximately 3.0% increase per 1-SD increase in HGI, but was not associated with postoperative length of stay (aMR 1.002, 95% CI 0.986–1.018; *P* = 0.808). No significant relationships were observed for the remaining secondary outcomes in the continuous models (all *P* > 0.05).

**Table 3:** Multivariable associations between hemoglobin glycation index and postoperative outcomes (continuous modeling)

Study outcome	Adjusted effect estimate (95% CI)	*P* value
Primary outcome		
Postoperative complications	1.068 (1.018–1.120)	0.007
Secondary outcomes		
Major complications	1.012 (0.946–1.082)	0.724
Pulmonary complications	1.016 (0.922–1.119)	0.747
Abdominal complications	0.969 (0.896–1.047)	0.426
Urinary complications	1.155 (1.024–1.301)	0.018
Infection complications	1.045 (0.970–1.126)	0.594
Cardiac complications	0.986 (0.874–1.112)	0.825
Neurological complications	1.019 (0.904–1.147)	0.757
Deep vein thrombosis	1.126 (1.005–1.259)	0.039
In-hospital mortality	0.790 (0.699–0.890)	0.001
LoS,days	1.030 (1.017–1.043)	0.001
Postoperative LoS,days	1.002 (0.986–1.018)	0.808
ICU admission	0.967 (0.926–1.009)	0.126
ICU LoS,hours	0.896 (0.758–1.058)	0.194

Adjusted estimates were derived from multivariable regression models including demographics, comorbidities, surgical characteristics, and the van Walraven–weighted Elixhauser comorbidity score, as specified in Methods. For binary outcomes, estimates are adjusted ORs. For length-of-stay outcomes, estimates are aMRs from generalized linear models with a log link. ICU length of stay was analyzed among patients admitted to the ICU. HGI was modeled as a continuous variable per 1-standard deviation increase

*aMRs* adjusted mean ratios, *CI* confidence interval, *ORs* odds ratios

### Quartile-based analyses (Q2 as reference)

To facilitate clinical interpretation and probe potential nonlinearity, HGI was additionally modeled in quartiles with Q2 as the reference (Table 4). The second quartile (Q2) was selected as the reference group because it exhibited the lowest incidence of outcome events in the unadjusted analysis (Table 2), and thus represented the lowest-risk category. Compared with Q2, both Q1 and Q4 were associated with higher odds of postoperative complications (Q1 OR 1.188, P = 0.024; Q4 OR 1.221, *P* = 0.016), whereas Q3 did not reach statistical significance (P = 0.068). Q1 was further associated with higher odds of major complications (OR 1.289, *P* = 0.033), pulmonary (OR 1.770, *P* = 0.006) and abdominal complications (OR 1.413, *P* = 0.009), infectious complications (OR 1.372, *P* = 0.029), ICU admission (OR 1.353, *P* = 0.001), and in-hospital mortality (OR 2.753, *P* = 0.001). In Q4, significant associations were observed for pulmonary complications (OR 1.936, *P* = 0.003), urinary complications (OR 2.022, *P* = 0.010), infectious complications (OR 1.695, *P* = 0.001), and ICU admission (OR 1.150, *P* = 0.049), while deep vein thrombosis was not significantly different across quartiles. For length-of-stay outcomes, compared with Q2, total hospital length of stay was longer in Q1 (aMR 1.055; + 5.5%), Q3 (aMR 1.108; + 10.8%), and Q4 (aMR 1.188; + 18.8%), and postoperative length of stay showed a similar pattern (Q1 aMR 1.119; + 11.9%; Q3 aMR 1.110; + 11.0%; Q4 aMR 1.172; + 17.2%). Among patients admitted to the ICU, ICU length of stay was substantially longer in Q1 (aMR 1.738; + 73.8%), while estimates for Q3 and Q4 were directionally higher but not statistically significant.

**Table 4:** Adjusted associations between hemoglobin glycation index quartiles and postoperative outcomes

Study outcome	HGI group	Adjusted effect estimate (95% CI)	*P* value
**Primary outcome**			
**Postoperative complications**	Q1	1.188 (1.023–1.379)	0.024
	Q2	Ref	
	Q3	1.152 (0.990–1.342)	0.068
	Q4	1.221 (1.038–1.436)	0.016
**Secondary outcomes**			
**Major complications**	Q1	1.289 (1.023–1.631)	0.033
	Q2	Ref	
	Q3	0.972 (0.752–1.256)	0.826
	Q4	1.211 (0.940–1.566)	0.141
**Pulmonary complications**	Q1	1.770 (1.191–2.686)	0.006
	Q2	Ref	
	Q3	1.654 (1.080–2.572)	0.022
	Q4	1.936 (1.263–3.024)	0.003
**Abdominal complications**	Q1	1.413 (1.092–1.838)	0.009
	Q2	Ref	
	Q3	1.097 (0.824–1.463)	0.525
	Q4	1.176 (0.877–1.582)	0.282
**Urinary complications**	Q1	1.591 (0.961–2.719)	0.079
	Q2	Ref	
	Q3	1.760 (1.045–3.044)	0.037
	Q4	2.022 (1.197–3.528)	0.010
**Infection complications**	Q1	1.372 (1.037–1.828)	0.029
	Q2	Ref	
	Q3	1.213 (0.896–1.648)	0.213
	Q4	1.695 (1.259–2.299)	0.001
**Cardiac complications**	Q1	1.091 (0.720–1.668)	0.684
	Q2	Ref	
	Q3	0.916 (0.589–1.430)	0.699
	Q4	1.044 (0.667–1.648)	0.853
**Neurological complications**	Q1	1.089 (0.716–1.673)	0.693
	Q2	Ref	
	Q3	1.133 (0.736–1.758)	0.573
	Q4	0.951 (0.602–1.518)	0.833
**Deep vein thrombosis**	Q1	0.949 (0.641–1.412)	0.795
	Q2	Ref	
	Q3	1.006 (0.680–1.495)	0.976
	Q4	1.146 (0.768–1.725)	0.507
**In-hospital mortality**	Q1	2.753 (1.532–5.300)	0.001
	Q2	Ref	
	Q3	1.031 (0.474–2.261)	0.938
	Q4	1.615 (0.821–3.339)	0.178
**LoS,days**	Q1	1.055(1.018–1.093)	0.003
	Q2	Ref	
	Q3	1.108(1.069–1.148)	0.001
	Q4	1.188(1.142–1.235)	0.001
**Postoperative LoS,days**	Q1	1.119(1.069–1.171)	0.001
	Q2	Ref	
	Q3	1.11(1.06–1.161)	0.001
	Q4	1.172(1.115–1.233)	0.001
**ICU admission**	Q1	1.353 (1.186–1.544)	0.001
	Q2	Ref	
	Q3	1.006 (0.878–1.153)	0.932
	Q4	1.150 (1.001–1.323)	0.049
**ICU LoS,hours**	Q1	1.738 (1.239–2.439)	0.001
	Q2	Ref	
	Q3	1.018 (0.804–1.290)	0.880
	Q4	1.286 (0.952–1.735)	0.101

Multivariable models were adjusted for the same covariates as in Table 3. HGI was categorized into quartiles with Q2 as the reference group. For binary outcomes, estimates are adjusted ORs. For length-of-stay outcomes, estimates are aMRs from generalized linear models with a log link. ICU length of stay was analyzed among patients admitted to the ICU

### Restricted cubic spline analyses (overall cohort)

**Fig. 3:**
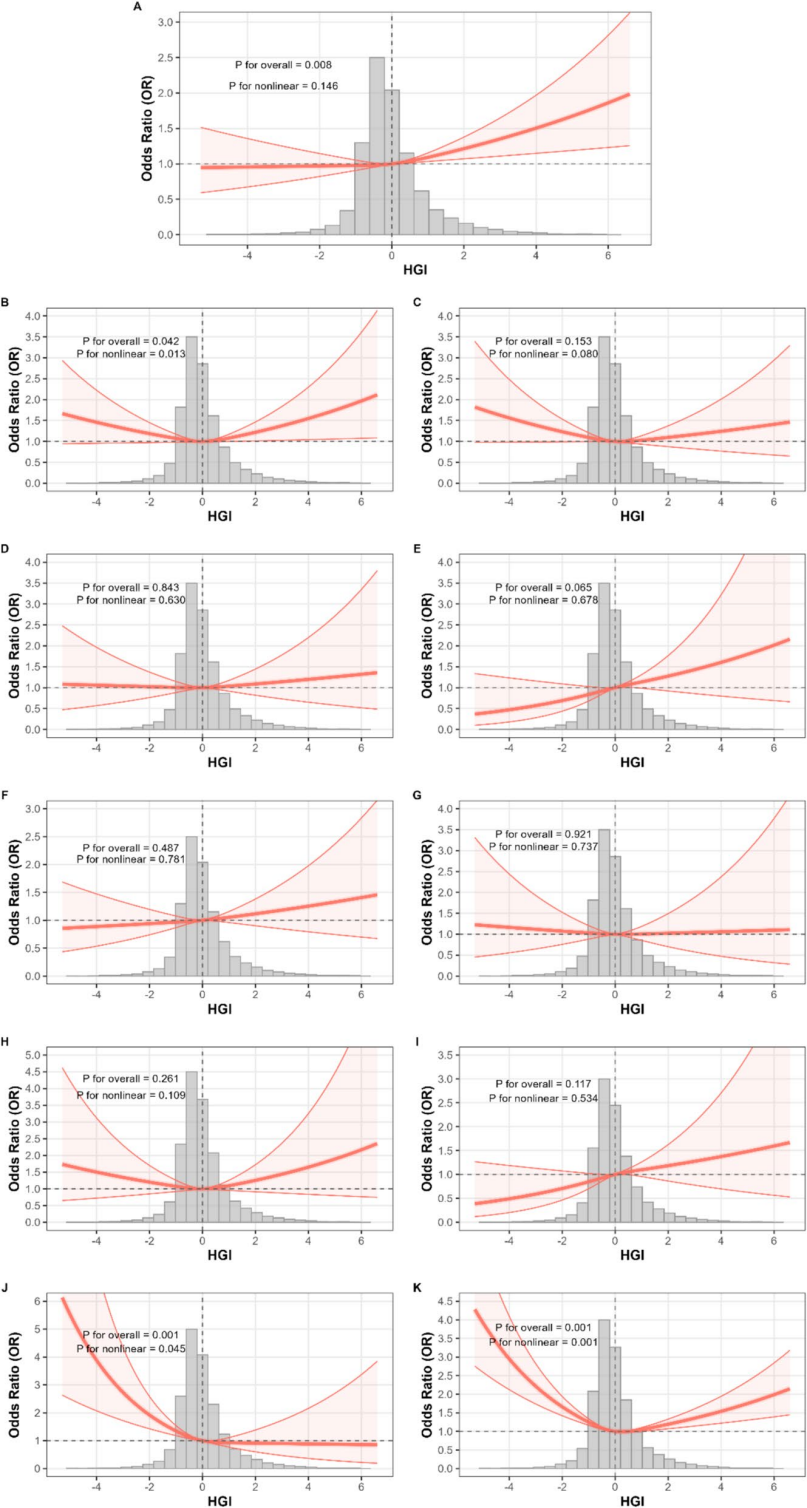
Adjusted associations between preoperative hemoglobin glycation index and postoperative outcomes. Restricted cubic spline models were used to evaluate the (non)linear associations between preoperative hemoglobin glycation index (HGI) and postoperative outcomes after non-cardiac surgery. Solid lines represent adjusted odds ratios (ORs), and shaded areas represent 95% confidence intervals. The vertical dashed line indicates the reference value of HGI = 0, and the horizontal dashed line indicates OR = 1. Gray histograms display the distribution of HGI in the study cohort. Panels: **A** Overall postoperative complications; **B** Major complications (Clavien–Dindo grade ≥ III); **C** Abdominal complications; **D** Pulmonary complications; **E** Urinary complications; **F** Infectious complications; **G** Cardiac complications; **H** Neurological complications; **I** Deep vein thrombosis; **J** In-hospital mortality; **K** ICU admission

Restricted cubic spline analyses for all outcomes are presented in Fig. 3A–K. Restricted cubic splines were then fitted to formally test for nonlinearity across the continuous HGI range (reference HGI = 0). In spline models, postoperative complications were associated with HGI overall (*P* for overall = 0.008) without evidence of nonlinearity (*P* for nonlinear = 0.146; Fig. 3A). Major complications showed significant nonlinearity (*P* for overall = 0.042; *P* for nonlinear = 0.013; Fig. 3B). In-hospital mortality and ICU admission also demonstrated nonlinear relationships (mortality: *P* for overall = 0.001; *P* for nonlinear = 0.045; Fig. 3J; ICU admission: *P* for overall = 0.001; *P* for nonlinear = 0.001; Fig. 3K). No significant overall associations were detected for abdominal (Fig. 3C), pulmonary (Fig. 3D), urinary (Fig. 3E), infectious (Fig. 3F), cardiac (Fig. 3G), neurological complications (Fig. 3H), or deep vein thrombosis (Fig. 3I) (all *P* for overall > 0.05).

Across modeling strategies, these findings suggest outcome-specific patterns of dose response: overall postoperative complications showed a broadly monotonic increase across the HGI range, whereas major complications, ICU admission, and in-hospital mortality displayed clear nonlinear patterns.

### Subgroup analyses and sensitivity analyses

**Fig. 4:**
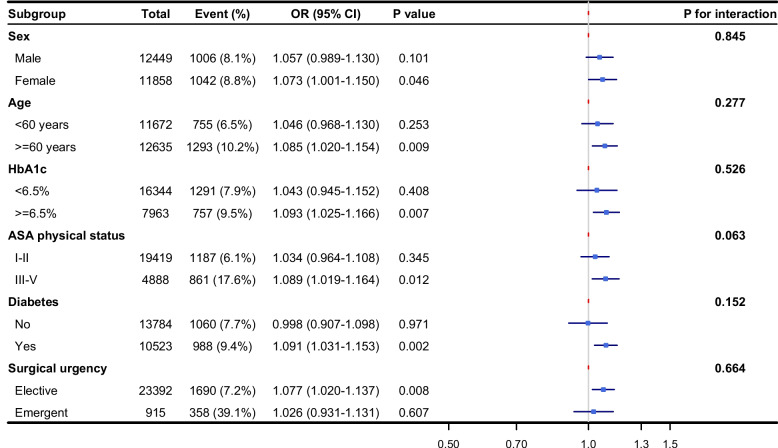
Subgroup analyses of the association between hemoglobin glycation index and postoperative complications. Forest plots show adjusted odds ratios (ORs) and 95% confidence intervals (CIs) for the association between hemoglobin glycation index (per 1-SD increase) and postoperative complications across prespecified subgroups, including sex, age, HbA1c level, ASA physical status, diabetes status, and surgical urgency. *P* values for interaction were calculated to assess potential effect modification across subgroups. Models were adjusted for the same covariates as in the primary multivariable analysis

Forest-plot subgroup analyses (Fig. 4) showed that the association between HGI and postoperative complications was broadly consistent across prespecified strata. Statistically significant associations were observed in female patients, patients aged ≥ 60 years, those with HbA1c ≥ 6.5%, patients with ASA physical status III–V, patients with diabetes, and those undergoing elective surgery. No statistically significant effect modification was detected across prespecified subgroups, although ASA physical status showed a borderline interaction (*P* for interaction = 0.063). Post-hoc power analyses of interaction tests revealed substantial variability across subgroups. For most subgroups, the estimated power to detect interaction effects was modest, and was particularly low in smaller subgroups such as emergent surgery (Supplementary Table 4). These findings suggest that the absence of statistically significant interaction should be interpreted cautiously, as limited statistical power may have reduced the ability to detect true effect modification.

**Fig. 5:**
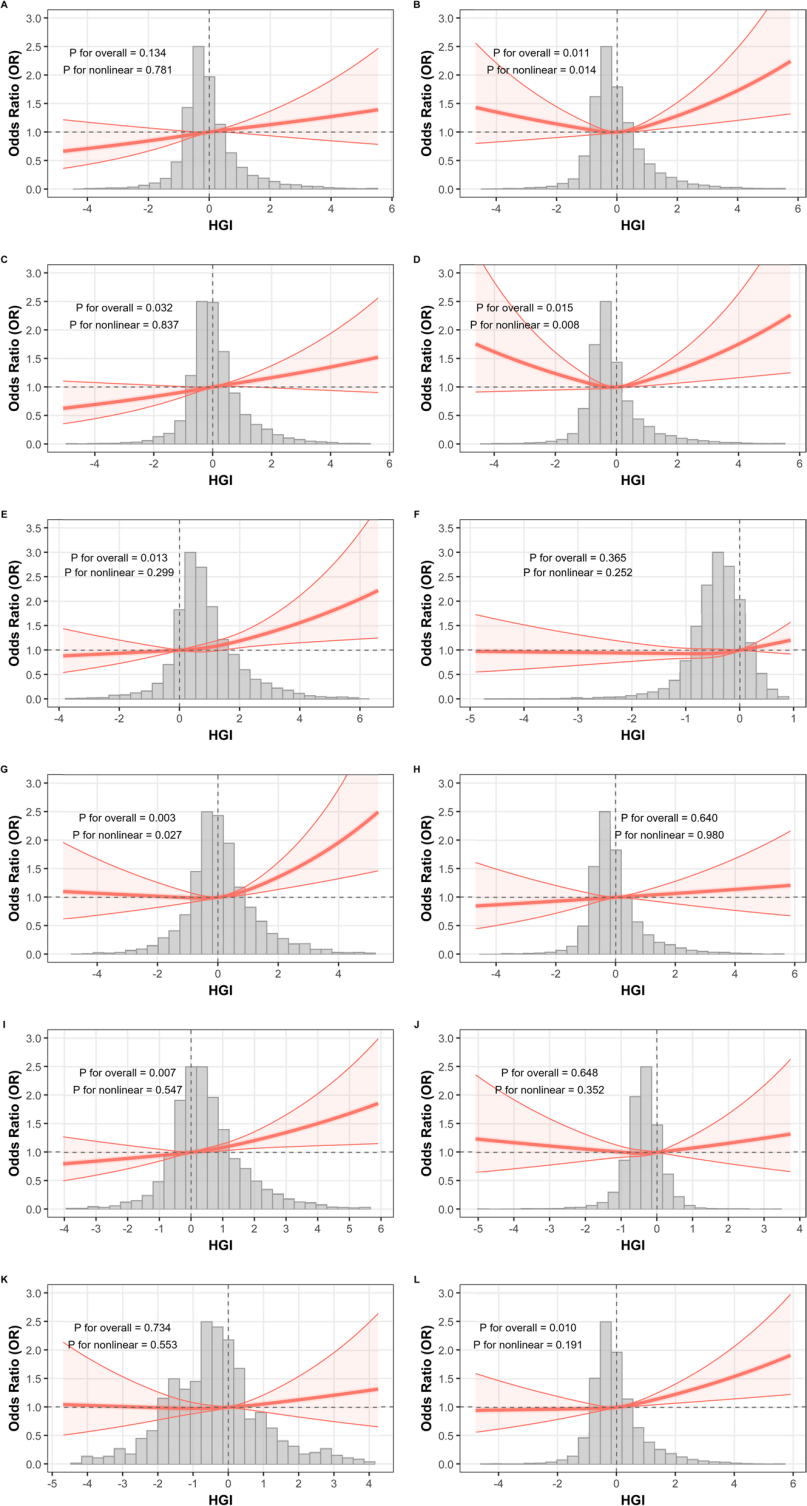
Subgroup-specific nonlinear associations between preoperative hemoglobin glycation index and postoperative complications. Restricted cubic spline models were used to evaluate subgroup-specific associations between preoperative hemoglobin glycation index (HGI) and postoperative complications. Solid lines represent adjusted odds ratios (ORs), and shaded areas represent 95% confidence intervals. The vertical dashed line indicates the reference value of HGI = 0, and the horizontal dashed line indicates OR = 1. Gray histograms show the distribution of HGI within each subgroup. Panels: **A** Female; **B** Male; **C** Age ≥ 60 years; **D** Age < 60 years; **E** HbA1c ≥ 6.5%; **F** HbA1c < 6.5%; **G** ASA III–V; **H** ASA I–II; **I** Diabetic patients; **J** Nondiabetic patients; **K** Emergent surgery; **L** Elective surgery

Subgroup-specific RCS analyses are shown in Fig. 5A–L. A significant nonlinear association was observed in male patients (*P* for overall = 0.011; *P* for nonlinear = 0.014; Fig. 5B), patients aged < 60 years (*P* for overall = 0.015; *P* for nonlinear = 0.008; Fig. 5D), and patients with ASA III–V (*P* for overall = 0.003; *P* for nonlinear = 0.027; Fig. 5G). In contrast, associations were weaker and not statistically significant in female patients (Fig. 5A), those with HbA1c < 6.5% (Fig. 5F), ASA I–II (Fig. 5H), and non-diabetic patients (Fig. 5J). The association was evident among elective surgeries (*P* for overall = 0.010; Fig. 5L) but not among emergent surgeries (Fig. 5K). No significant associations were observed in other subgroups (Fig. 5C, E, I). Collectively, these subgroup analyses suggested that the HGI–risk relationship may be more pronounced and more likely to exhibit nonlinearity in selected higher-risk populations.

In sensitivity analyses replacing binary diabetes status with a 3-level glycemic-status variable, the association between HGI and postoperative complications remained materially unchanged. Specifically, the adjusted OR per 1–SD increase in HGI was 1.069 (95% CI, 1.019–1.121;* P* = 0.006), and quartile-based estimates were similar to the primary analysis (Supplementary Table 5–7).

## Discussion

In this retrospective cohort of 24,307 adults undergoing non-cardiac surgery, postoperative complications occurred in 8.4%. Across complementary multivariable approaches, higher preoperative HGI was associated with increased odds of overall postoperative complications, with additional associations observed for urinary complications (primarily acute kidney injury and related diagnoses) and longer total hospital stay. In contrast, categorical and spline models indicated that severe outcomes (major complications, ICU admission, and in-hospital mortality) displayed outcome-specific departures from linearity, with the lowest risks generally observed in the mid-range HGI group (Q2) and higher risks at the extremes—particularly at very low HGI levels. These findings suggest that different postoperative endpoints may capture different dimensions of perioperative metabolic vulnerability. One possible explanation is that overall postoperative complications represent a heterogeneous composite endpoint with relatively high event frequency, making its association with HGI more likely to appear as an average monotonic risk gradient across the population. By contrast, severe outcomes such as major complications, ICU admission, and in-hospital mortality are clinically more extreme and may be driven disproportionately by patients at the tails of the HGI distribution. Accordingly, risk for these endpoints may not rise uniformly across the entire HGI range, but instead become more apparent only at extreme HGI values.

HGI operationalizes interindividual discordance between HbA1c and contemporaneous glycemia and is commonly interpreted as a marker of glycation propensity. Prior studies have linked higher HGI to microvascular complications, adverse cardiovascular events, and kidney injury [25–30], although findings remain heterogeneous and the perioperative predictive value of HGI is still debated [31–33]. In our cohort, HbA1c increased stepwise across HGI quartiles, while fasting glucose showed a non-monotonic pattern, consistent with the residual-based definition of HGI and supporting the notion that HGI captures information beyond absolute glycemic levels.

Several biologically plausible pathways may connect higher HGI to postoperative morbidity. A higher HGI implies higher-than-expected HbA1c for a given glucose level and may reflect enhanced nonenzymatic glycation and downstream accumulation of advanced glycation end products (AGEs). Although AGEs were not directly measured in the present study, prior experimental and clinical studies have shown that increased glycation burden is associated with AGE accumulation, oxidative stress, endothelial dysfunction, and inflammation, which may impair microcirculatory perfusion, host defense, and tissue repair—processes central to postoperative complications [34–37]. Therefore, the proposed AGE-related mechanism should be interpreted as a biologically plausible hypothesis rather than a directly demonstrated pathway in the current cohort. Higher HGI has also been associated with broader metabolic dysregulation, including abnormalities in lipid and protein metabolism [38], potentially amplifying vulnerability to perioperative stress. The numerically stronger association in patients with ASA III–V may reflect greater physiologic vulnerability, but this finding should be interpreted cautiously given the absence of statistically significant interaction.

Another important consideration relates to the definition of diabetes status. In the primary analysis, diabetes was modeled as a binary variable based on documented diagnosis, which may not fully capture the heterogeneity of glycemic burden. First, information on diabetes duration was unavailable; patients with similar HbA1c levels but different disease durations may differ substantially in cumulative metabolic injury and perioperative risk. Second, the “non-diabetic” group may include individuals with undiagnosed diabetes or prediabetes, potentially leading to misclassification bias. To address this concern, we performed a sensitivity analysis in which glycemic status was reclassified using both documented diagnosis and laboratory criteria. Reassuringly, the association between HGI and postoperative complications remained materially unchanged, suggesting that misclassification of diabetes status is unlikely to fully explain our findings. Nevertheless, residual confounding related to glycemic history cannot be entirely excluded.

In-hospital mortality showed a distinct, nonlinear relationship with HGI. While linear modeling suggested an inverse association, spline and quartile analyses revealed an L-shaped pattern with excess risk concentrated at very low HGI values, consistent with prior observations in selected settings. Low HGI may reflect disproportionately elevated fasting glucose relative to HbA1c—compatible with acute stress hyperglycemia and higher illness severity [39] —or may be influenced by non-glycemic determinants of HbA1c (e.g., altered erythrocyte turnover), both of which could contribute to higher short-term mortality risk [40]. The enrichment of emergency surgeries in the low-HGI group further supports the possibility that low HGI functions as an acuity phenotype marker rather than a benign glycemic state.

In our cohort, the lowest HGI group (Q1) had the highest proportion of emergency surgery (5.7% vs. 1.9%–3.3% in the other quartiles), the highest crude in-hospital mortality (1.7% vs. 0.2%–0.6%), more ICU admissions (17% vs. 10% in Q2), longer ICU stay, and higher rates of major, pulmonary, abdominal, and infectious complications. Patients undergoing emergency surgery are more likely to involve trauma, acute bleeding, and physiological instability. An additional consideration relates to the potential impact of acute physiological states on HbA1c and, consequently, HGI estimation. Acute blood loss and perioperative stress may accelerate erythropoiesis and reduce the average lifespan of circulating red blood cells, leading to spuriously lower HbA1c values independent of true glycemic exposure. In such scenarios, HGI may be systematically shifted toward negative values. Therefore, the observed excess in-hospital mortality in Q1 may, at least in part, reflect residual confounding from acute illness severity and altered erythrocyte turnover, rather than a direct effect of a metabolic phenotype. This limitation should be considered when interpreting the nonlinear association between HGI and mortality. Therefore, the association between very low HGI and in-hospital mortality should be interpreted cautiously, as it may partly reflect residual confounding from acute illness severity and altered erythrocyte kinetics rather than a direct adverse effect of a low-HGI metabolic phenotype., particularly in the perioperative setting.

Clinically, HGI may add perioperative risk information by flagging patients with marked HbA1c–glucose discordance. The primary outcome increased with higher HGI, whereas major complications, ICU admission, and mortality were most informative at the extremes, suggesting that HGI may be most useful for identifying high-risk phenotypes rather than as a purely linear biomarker. Prospective validation and evaluation of incremental predictive value beyond HbA1c and fasting glucose are needed before clinical implementation.

Several limitations merit consideration. First, an important methodological issue is that HGI depends on the regression equation used to predict HbA1c from fasting glucose. Our derivation equation differed from previously published formulas, including those reported in the REACTION cohort and the MIMIC-IV critical coronary artery disease cohort [26, 27]. This variation likely reflects differences in source population, disease spectrum, assay conditions, glucose units, and clinical context. Therefore, we used a cohort-specific equation for the primary analysis, which is consistent with the conceptual basis of HGI as a residualized, population-dependent measure rather than a universally transportable absolute biomarker. Second, the retrospective design precludes causal inference and residual confounding is likely; perioperative antibiotic prophylaxis, diabetes duration, perioperative glucose trajectories, intraoperative glucose management, and relevant medications were unavailable. In addition, preoperative inflammatory and nutritional markers (e.g., C-reactive protein, albumin), which may reflect oxidative stress and systemic vulnerability related to glycation processes, were not available in our dataset. The absence of these variables may limit mechanistic interpretation of the observed associations. Third, HGI was derived using a single fasting glucose value rather than mean blood glucose, which is susceptible to short-term variability (including stress and timing effects). Fourth, restricting analyses to patients with both HbA1c and fasting glucose may introduce selection bias, and outcome ascertainment based on discharge diagnoses may lead to misclassification. Another limitation is that no formal adjustment for multiple comparisons was applied to secondary, subgroup, or sensitivity analyses, which may increase the risk of type I error. Therefore, these findings should be considered exploratory and hypothesis-generating rather than confirmatory, and require validation in future studies. We did not apply formal correction because these analyses were prespecified and exploratory; overly conservative adjustment might obscure potentially meaningful signals. Despite these limitations, the large sample size and concordant signals across continuous, categorical, spline, and subgroup analyses support the hypothesis that HbA1c–glucose discordance captures clinically relevant vulnerability in surgical patients.

## Conclusions

In this large retrospective cohort of adults undergoing non-cardiac surgery under general anesthesia, preoperative HGI was independently associated with postoperative complications. Categorical and spline analyses indicated outcome-specific nonlinearity, with the lowest risk generally observed in the mid-range HGI group and higher risks at extreme HGI values for selected endpoints, including major complications, ICU admission, and in-hospital mortality. These findings suggest that HbA1c–glucose discordance, captured by HGI, may provide incremental information for perioperative risk stratification beyond HbA1c and fasting plasma glucose. Prospective multicenter studies are needed to validate generalizability and to determine whether incorporating HGI into perioperative risk assessment improves clinical decision-making and outcomes.

## Supplementary Information

Supplementary Material 1.

## Data Availability

The data that support the findings of this study are available from the corresponding author upon reasonable request, subject to institutional ethical approval.

## Abbreviations

AGEs: Advanced glycation end products
aMR: Adjusted mean ratio
ASA: American Society of Anesthesiologists
BMI: Body mass index
CI: Confidence interval
DVT: Deep-vein thrombosis
FPG: Fasting plasma glucose
HbA1c: Hemoglobin A1c
HGI: Hemoglobin glycation index
ICD: International Classification of Diseases
ICU: Intensive Care Unit
IQR: Interquartile range
MAR: Missing at random
OR: Odds ratio
PUMCH: Peking Union Medical College Hospital
SD: Standard deviation
RCS: Restricted cubic spline
VIF: Variance inflation factor
